# Associations of training to assist a suicidal person with subsequent quality of support: results from a national survey of the Australian public

**DOI:** 10.1186/s12888-018-1722-y

**Published:** 2018-05-18

**Authors:** Anthony F. Jorm, Angela Nicholas, Jane Pirkis, Alyssia Rossetto, Nicola J. Reavley

**Affiliations:** Centre for Mental Health, Melbourne School of Population and Global Health, The University of Melbourne, 207 Bouverie St, Carlton, VIC 3010 Australia

**Keywords:** Suicide, Mental health first aid, Gatekeepers

## Abstract

**Background:**

When a person is in severe distress, people in their social network can potentially take action to reduce the person’s suicide risk. The present study used data from a community survey to examine whether people who had received training in how to assist a person at risk of suicide had higher quality intentions and actions to provide support.

**Methods:**

A national telephone survey was carried out with 3002 Australian adults on attitudes and intentions toward helping someone in severe distress or at risk of suicide as well as actions taken. Participants were asked about their intentions to assist a hypothetical person in a vignette and about any actions they took to assist a family member or friend in distress over the previous 12 months. Participants were also asked whether they had received professional training, Mental Health First Aid training or other training in how to assist a person at risk of suicide.

**Results:**

Responses covered ten intentions/actions that were recommended in guidelines for the public on how to support a suicidal person and 5 that were recommended against in the guidelines. Scales were created to measure positive and negative intentions to act and positive and negative actions taken. All three types of training were associated with greater positive intentions and actions, and with lesser negative intentions. These associations were largely due to a greater willingness of those trained to talk openly about suicide with a person in distress.

**Conclusions:**

Training in how to support a person at risk of suicide is associated with better quality of support. Such training merits wider dissemination in the community.

**Electronic supplementary material:**

The online version of this article (10.1186/s12888-018-1722-y) contains supplementary material, which is available to authorized users.

## Background

While mental health and primary care services can play an important role in detecting suicide risk and acting to reduce it, many people at risk of suicide are not in immediate contact with services. Psychological autopsy studies show that less than half of people who die by suicide are in contact with primary care services in the month before their death, and the rate of contact is even lower for specialist mental health care [1, 2]. For this reason, members of a suicidal person’s social network may be well placed to detect and act to reduce the person’s suicide risk. However, there are a number of barriers to members of a person’s social network taking on this role. While about one-third to a half of people who die by suicide explicitly communicate their intent to family members [3], in other cases the indicators of suicidal intent may be unclear and misread by the person’s family and friends [4, 5], and thereby not prompt preventive action. People in the social network may also not feel comfortable raising the issue of suicide with the person and alerting others in the social network [4]. They may also respond in a dismissive or disapproving way to the person’s expressions of suicidal feelings [6], thereby shutting down communication.

There is a need to determine what actions members of a suicidal person’s social network can take that are likely to be helpful and also what actions should be avoided. However, it is not feasible to test the preventive effects of specific actions by the public using experimental methods. For this reason, expert consensus has been used to develop suicide first aid guidelines for the public using the Delphi method. Kelly and colleagues [7] recruited 22 professionals, 10 people who had been suicidal in the past and 6 carers of people who had been suicidal in the past and presented them with 114 statements about how to assist someone who is thinking about suicide. These statements were sourced through a systematic search of both professional and lay literature. Thirty of these statements were endorsed at a high level and formed into guidelines. Subsequently, Ross and colleagues [8] re-developed the guidelines using two expert panels, comprising 41 suicide prevention professionals and 35 consumer advocates. The panelists rated 436 statements and endorsed 164 which were used to form updated guidelines. These guidelines provide a standard for improving the support that members of the public provide to suicidal persons in their social network.

When judged against these guidelines, community survey data reveal limitations in the public’s ability to act effectively to prevent suicide. In two Australian national surveys, respondents were shown a vignette of a person with depression and suicidal thoughts and asked what they would do if the person was someone they knew and cared about [9, 10]. Coding of open-ended responses showed that while many would listen to the person, provide support and encourage professional help-seeking, very few would assess the person’s risk of suicide or act to reduce this.

A range of gatekeeper training programs have been developed to improve the response of the public and professionals to suicidal persons. A review of these programs concluded that gatekeeper training can improve knowledge, beliefs/attitudes, self-efficacy, and reluctance to intervene, but transfer to actual intervention behaviour is largely unstudied [11]. In Australia, a number of training programs have been rolled out to improve the public’s ability to assist suicidal persons. Probably the most widespread is Mental Health First Aid (MHFA), which is a 12–14 h course training members of the public in how to provide initial assistance to someone developing a mental health problem or in a mental health crisis, including helping a person with suicidal thoughts or behaviours [12]. MHFA training has been received by over 2% of the Australian population. A meta-analysis of trials evaluating MHFA showed improvements in knowledge, stigmatizing attitudes and helping behaviours towards people with mental health problems [13]. However, there has not been any specific evaluation of the impact of MHFA training on support given to suicidal persons. Other common programs in Australia are QPR (Question, Persuade, and Refer) and Applied Suicide Intervention Skills Training (ASIST). QPR is a 1.5–2 h course specifically on suicide prevention. Evaluations of QPR have shown improvements in knowledge, self-efficacy and helping behaviour [14]. ASIST is a 2-day program aimed at both professionals and the public. Evaluations of ASIST have found improvements in knowledge, confidence and intervention skills, but mixed results on intervention behaviour [15].

The present study aimed to use Australian national survey data to investigate associations between training in how to assist a suicidal person and actions taken on suicide. The survey investigated Australian community members’ attitudes, intentions and behaviours toward helping someone in severe distress or at risk of suicide. As part of this survey, participants were asked about their intentions to assist a hypothetical person in a vignette and about any actions they took to assist a family member or friend in distress over the previous 12 months. Participants were asked about intentions and actions that were either recommended or not recommended in the expert-consensus guidelines developed by Ross and colleagues [8]. Participants were also asked about any training or course they had taken in how to help someone who is suicidal, allowing a comparison between those who had received various types of training and those untrained. It was hypothesized that people who had received training would have higher quality intentions and actions.

## Methods

### Participants

The survey was commissioned by *beyondblue*, which is an Australian, non-government non-profit organization working to address issues associated with depression and anxiety disorders. The survey was conducted by Roy Morgan Research Ltd. in March 2017. The sample was drawn by a process of random digit dialing of both landlines and mobile telephones covering the whole of Australia. Up to six calls per number were made to establish contact. Interviewers ascertained whether there were residents in the household aged 18 or over and, if there were multiple, selected one for interview using the next-birthday method. Oral consent was obtained from all respondents before commencing the interviews. Computer-assisted telephone interviews were carried out with 3002 people. There are a number of ways to calculate survey response rates. For this survey, the American Association for Public Opinion Research response rate [16] was 3.1% and the simple response rate was 12.2%.

### Measures

The survey interview covered sociodemographic characteristics, intentions and confidence to help a person in distress, barriers and enablers, actual helping behaviour, the participant’s own suicidal thoughts, help received, attitudes to suicide, exposure to suicide, training in suicide prevention and exposure to suicide prevention messages in the media. The full interview is given in Additional file 1. Only the measures of specific relevance to the aims of the present paper are described in detail below.

#### Sociodemographics

Participants were asked questions about sociodemographic characteristics, which were coded as follows for the analyses reported here: female gender, age group (18–30, 31–59, 60+), mainly speak a language other than English, education with Bachelor’s degree or above and non-urban location.

#### Helping intentions

Helping intentions were assessed in relation to one of six vignettes of distressed persons that were randomly assigned to participants. The vignettes covered male or female versions of three scenarios: a person with distress and adverse life events, a person with distress and adverse life events but no overt suicidality (“John/Jenny says he/she feels he/she will never be happy again and believes his/her family would be better off without him/her”), and a person with distress and adverse life events with overt suicidality (“John/Jenny says he/she feels s/he will never be happy again and believes his/her family would be better off without him/her. You run into a friend of John’s/Jenny’s. S/he tells you that John/Jenny told him/her he/she feels desperate and has been thinking of ways to end his/her life”). The six scenarios are given in Additional file 1.

Participants were then asked “How likely is it that you would take the following actions with John/Jenny?” Very unlikely, Unlikely, Neither likely nor unlikely, Likely, Very likely. The actions presented were: “Ask about how he/she is feeling; Listen to John’s/Jenny’s problems without judgement; Remind him/her what he/she has going for himself/herself*; Ask how you can help; Try to solve John’s/Jenny’s problems*; Reassure John/Jenny that you know exactly how badly he/she feels*; Help make an appointment with a health professional – for example a GP or counsellor; Call a crisis line – for example, Lifeline; Go to an appointment with a professional with him/her – for example a GP; Ask if he/she has been thinking about killing himself/herself; If John/Jenny told me he/she was thinking about killing himself/herself, I would try to make him/her understand that suicide is wrong*; If John/Jenny told me he/she was thinking about killing himself/herself, I would ask if he/she has a means to kill herself/himself – for example, pills or a weapon; If John/Jenny told me he/she was thinking about killing himself/herself, I would listen to why he/she wants to die; I would tell him/her how much it will hurt his/her family and friends if he/she were to kill himself/herself*; I would ask if he/she has a plan for suicide – for example a date or how they will die”.

Ten of the items above are recommended by expert-consensus guidelines, while 5 are recommended against (the latter are asterisked above) [8]. The 10 recommended items were made into a Positive Intentions scale by averaging the ratings across items to give a score range from 1 (every item rated ‘very unlikely’) to 5 (every item rated ‘very likely’). Similarly, the 5 items recommended against were averaged to give a Negative Intentions scale from 1 to 5.

#### Helping behaviour

Participants were asked “In the last 12 months, has anyone in your family or close circle of friends experienced a similar level of distress to John/Jenny?” and “Did just one of your family or close friends experience this level of distress in the last 12 months, or more than one?”. If the participant knew more than one person, they were told: “Because you know more than one family member or close friend experiencing a similar level of distress, for the next few questions, I want you to think about the one you know BEST”. Participants were asked an open-ended question about what they did to help the person and then a series of questions about specific actions taken that paralleled the questions on intentions. The interviewer recorded ‘yes’ or ‘no’ for each of the 15 items listed above for measuring intentions.

As for the intentions items above, the 10 recommended items were made into a Positive Actions scale by summing the number of ‘yes’ responses to give a score range from 0 (no positive actions carried out) to 10 (all positive actions carried out). Similarly, the 5 items recommended against were summed to give a score range from 0 (no negative actions carried out) to 5 (all negative actions carried out).

#### Exposure to suicide

Participants were asked “Do you know anyone who has died by suicide?”, with responses recorded as yes or no.

#### Training received

Participants were asked “Have you ever completed any training or course in how to help someone who is suicidal?” The interviewer coded responses as professional training, MHFA, ASIST, QPR or other. The commissioning organization *beyondblue* is not associated with any of the training programs evaluated in the present study.

### Statistical analysis

Items concerning intentions and actions recommended or not recommended in expert-consensus guidelines were made into scales. Reliability of these scales was quantified with coefficient omega-total using the statistical package R [17].

The associations between type of training received and quality of intentions and actions were examined using simultaneous linear regression in IBM SPSS Statistics 22. Types of training (professional, MHFA, other) were coded as dichotomous variables and used as predictors of scale scores, with adjustment for type of vignette (dummy coded), sociodemographic characteristics and exposure to suicide. The sociodemographic variables and exposure to suicide were used as covariates because they all had associations (*P* < 0.05) with having received at least one type of training. Unstandardized regressions coefficients and their 95% CIs are reported for types of training. Effect sizes were measured using Cohen’s d by dividing unstandardized regression coefficients by the sample standard deviation, with values of 0.2, 0.5 and 0.8 being regarded as ‘small’ , ‘medium’ and ‘large’ respectively.

Where associations were found at *P* < 0.05 for types of training, post-hoc regression analyses were carried out to explore associations with individual intention and action items as the outcome variables. Linear regression was used for associations with the intention items (which were rated on a Likert scale) and binary logistic regression for the action items (which were yes/no). Because these exploratory analyses were post-hoc and involved multiple outcome variables, a conservative Bonferroni approach was used, with alpha divided by the number of items in a scale.

## Results

**Fig. 1:**
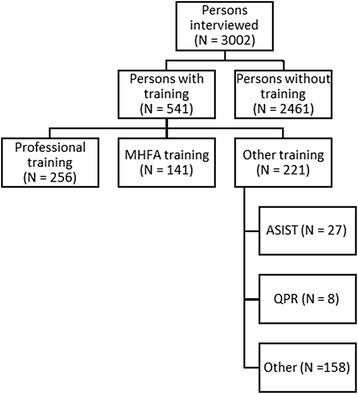
Breakdown of the sample according to training received

Table 1 shows the sociodemographic characteristics of the sample. Figure 1 shows the breakdown of the sample according to training received. Because the number of participants who had done ASIST and QPR was small), these were combined with the Other group. The categories of training overlapped, because some people had more than one type of training.

**Table 1:** Sociodemographic characteristics of the sample (*N* = 3002)

Characteristic	N (%)
Female gender	1785 (59.5%)
Aged 18–30	356 (11.9%)
Aged 31–59	1408 (46.9%)
Aged 60+	1238 (41.2%)
Bachelor’s degree or above	1215 (40.5%)
Non-urban location	1254 (41.8%)
Exposed to suicide	1839 (61.3%)

Table 2 shows the descriptive statistics on the Positive and Negative Intentions scales and the Positive and Negative Actions scales. This table also gives the omega reliability coefficients for the scales, with values generally being acceptable.

**Table 2:** Descriptive statistics and reliabilities (coefficient omega) for the intentions and actions scales

Scale	Mean (SD)	Range	Omega (interval)^a^	Omega (ordinal)^a^
Positive Intentions	4.02 (0.61)	1–5	0.80	0.85
Negative Intentions	3.77 (0.74)	1–5	0.68	0.74
Positive Actions	5.50 (2.29)	0–10	0.75	0.85
Negative Actions	2.71 (1.44)	0–5	0.64	0.76

^a^Omega-total assuming either interval or ordinal level of measurement

Table 3 shows the inter-correlations among the scales. It can be seen that positive intentions were more highly correlated with positive actions than with negative actions (0.44 vs 0.26). Conversely, negative intentions were more highly correlated with negative actions than with positive actions (0.54 vs 0.13). However, positive intentions were correlated 0.34 with negative intentions, and positive actions were correlated 0.51 with negative actions, even though one set is recommended by experts and the other recommended against, indicating a general tendency to intend to take action or not.

**Table 3:** Correlations among intentions and actions scales

Scale	Positive intentions	Negative intentions	Positive actions	Negative actions
Positive Intentions	1.00	0.34	0.44	0.26
Negative Intentions		1.00	0.13	0.54
Positive Actions			1.00	0.51
Negative Actions				1.00

For all correlations, *P* < 0.001

Of the 3002 participants, 1056 knew someone who was distressed in the past 12 months and 935 did something to support the person. Multiple binary regression analyses were carried out to see whether training was a predictor of knowing someone and, among the subgroup that did, whether support was provided. After adjusting for covariates, only MHFA training was associated with knowing someone (OR = 1.51, 95% CI 1.05–2.17, *P* = 0.026). There was no association of training with provision of support.

Multiple linear regression analyses were carried out to explore whether type of training predicted intention and action scale scores. The unstandardized coefficients after adjustment for covariates are shown in Table 4. Table 5 shows the corresponding values of Cohen’s d. All types of training were associated with greater positive intentions and actions, with similar effect sizes in the small-to-medium range. Similarly, all types of training were associated with lesser negative intentions, although the effect sizes were greater for professional training (small-to-medium) than for MHFA and other training (small). By contrast, all associations with negative actions were non-significant and less than small.

**Table 4:** Unstandardized regression coefficients (and 95% CIs) for associations between type of training and scales measuring intentions and actions^a^

Scale	Professional training	MHFA	Other training
Positive Intentions	0.24 (0.16, 0.31)	0.26 (0.16, 0.36)	0.21 (0.13, 0.29)
Negative Intentions	−0.25 (−0.34, − 0.16)	−0.15 (− 0.27, − 0.02)	− 0.16 (− 0.26, − 0.07)
Positive Actions	0.76 (0.26, 1.26)	0.98 (0.39, 1.57)	0.78 (0.29, 1.28)
Negative Actions	−0.22 (− 0.53, 0.10)	0.26 (− 0.11,0.64)	−0.16 (− 0.48, 0.16)

^a^Adjusted for sociodemographics, type of vignette presented and exposure to suicide

**Table 5:** Cohen’s d (and 95% CIs) for associations between type of training and scales measuring intentions and actions^a^

Scale	Professional training	MHFA	Other training
Positive Intentions	0.39 (0.26, 0.51)	0.43 (0.26, 0.59)	0.34 (0.21, 0.48)
Negative Intentions	−0.34 (− 0.46, − 0.22)	−0.20 (− 0.36, − 0.03)	−0.22 (− 0.35, − 0.09)
Positive Actions	0.33 (0.11, 0.55)	0.43 (0.17, 0.69)	0.34 (0.13, 0.56)
Negative Actions	−0.15 (− 0.37, 0.07)	0.18 (− 0.08, 0.44)	−0.11 (− 0.33, 0.11)

^a^Adjusted for sociodemographics, type of vignette presented and exposure to suicide

Table 6 shows post-hoc analyses of associations of training with specific Positive and Negative Intentions items, while Table 7 shows post-hoc analyses of associations with specific Positive Actions items. These significant associations are with items that involve explicit communication about suicide.

**Table 6:** Significant associations between type of training and specific intentions^a^

Type of training	Positive intentions that were more likely	Negative intentions that were less likely
Professional	Ask if he/she has been thinking about killing himself/herself. Ask if he/she has the means to kill himself/herself. Ask if he/she has a plan for suicide—for example a date or how they will die.	Reassure you know exactly how badly he/she feels. Would try to make him/her understand that suicide is wrong. Tell him/her how much it will hurt his/her family and friends if he/she were to kill himself/herself.
MHFA	Ask if he/she has been thinking about killing himself/herself. Ask if he/she has the means to kill himself/herself. Ask if he/she has a plan for suicide—for example a date or how they will die.	Would try to make him/her understand that suicide is wrong.
Other	Listen to problems without judgement Ask if he/she has been thinking about killing himself/herself. Ask if he/she has the means to kill himself/herself. Ask if he/she has a plan for suicide—for example a date or how they will die.	Would try to make him/her understand that suicide is wrong.

^a^Significant with Bonferroni-adjusted alpha of 0.05/15 = 0.003

**Table 7:** Significant associations between type of training and specific positive actions^a^

Type of training	Positive actions that were more likely
Professional	Ask if he/she has the means to kill himself/herself. Ask if he/she has a plan for suicide—for example a date or how they will die.
MHFA	Ask if he/she has been thinking about killing himself/herself. Ask if he/she has the means to kill himself/herself. Ask if he/she has a plan for suicide—for example a date or how they will die.
Other	Ask if he/she has the means to kill himself/herself. Ask if he/she has a plan for suicide—for example a date or how they will die.

^a^Significant with Bonferroni-adjusted alpha of 0.05/10 = 0.005

## Discussion

The findings show that training in how to help a suicidal person is associated with increased intentions to act in ways recommended by guidelines for the public, and decreased intentions to act in ways recommended against by the guidelines. All three types of training—professional, MHFA and other—had similar small-to-medium associations with positive intentions. However, the association with reduced negative intentions was small-to-medium for professional training, but only small for MHFA and other training. For actions to help a distressed person in the past 12 months, all types of training were associated with taking more actions recommended by the guidelines, with small-to-medium effect sizes. Associations with actions not recommended were not significant. When specific intentions and actions were examined, training was associated specifically with a greater willingness to talk openly about suicide with a distressed person. It should be noted when considering these findings that the associations with professional training were in relation to helping a family member or friend, rather than in the context of helping a client in a professional role.

We are not aware of any previous studies that have examined associations with suicide-relevant training in the context of a community survey. However, a US study used a similar approach in a large cross-sectional survey of behavioural health care staff [18]. This study found that staff who had received training in ASIST, QPR or other suicide-relevant training had greater suicide knowledge and confidence in working with suicidal persons. However, unlike the present study, the US study did not assess whether this greater knowledge and confidence was associated with behaviour.

The present study found associations between intentions to support a hypothetical person in a vignette and supportive actions to a family member or friend in the previous 12 months. The associations showed some specificity, with positive intentions correlating with positive actions and negative intentions with negative actions. While the associations were measured cross-sectionally in the current study, they support the findings from longitudinal studies that mental health first aid intentions predict subsequent mental health first aid actions [19, 20]. These findings indicate that suicide helping intentions can be used as a proxy short-term outcome where it is not feasible to measure behaviour, e.g. at a post-test assessment after a training course.

The major limitation of the study is that the data are cross-sectional, limiting causal inference. Although a variety of potential confounders were adjusted for, it is possible that there are other unmeasured differences between the groups that were not. We also lacked data on how long ago the training was received and, in many cases, information about what the content of the training was. If the training was within the past year, then it is possible that it followed rather than preceded any reported behaviour. The only specific type of training we can draw conclusions on is MHFA, which is a broader type of training in how to assist people developing mental health problems or in mental health crisis situations, with assisting a suicidal person being only one component. In fact, the present study is the first to examine suicide-specific outcomes of MHFA training and adds to the evidence from trials that such training increases knowledge, reduces stigmatizing attitudes and improves supportive behaviours towards people with mental health problems [13].

Other limitations are the low response rate and possible biases in the sample. We found that 4.7% of the sample reported having received MHFA training, whereas the population estimate of adults having done MHFA training is 2.6%, indicating an over-representation of people with an interest in mental health.

On the other hand, the methods used in the present study have some strengths. The study examined the effects of training in a real-life community context. In trials to evaluate suicide-relevant training, the measures used generally only cover short-term changes and are often transparent in their purpose and thus potentially subject to biased reporting to please the researcher. In the current study, there was no obvious connection between the purposes of the study and any training received. Participants were told that the study was on “what Australian adults understand about recognising and assisting an individual in severe distress”, with the question on training being asked towards the end of the interview.

## Conclusions

The findings show that training in how to help someone who is suicidal is associated with better quality intentions and actions towards a distressed person in the social network, in particular a greater willingness to talk openly about suicide. The magnitude of the association was similar for short courses and professional training. If these benefits can be confirmed in controlled trials, it would indicate that such courses merit wider dissemination in the community to increase the support provided to suicidal persons and to reduce the risk of suicide.

## Additional file

Additional file 1: Full interview schedule. (DOCX 51 kb)

## Abbreviations

ASIST: Applied Suicide Intervention Skills Training
MHFA: Mental Health First Aid
QPR: Question, Persuade, Refer
SPSS: Statistical Package for the Social Sciences
