# Supplementary material for: Associations of training to assist a suicidal person with subsequent quality of support: results from a national survey of the Australian public
Source: BMC Psychiatry. 2018 May 18;18:132. doi: 10.1186/s12888-018-1722-y (PMC5960111; doi:10.1186/s12888-018-1722-y)
Supplement: Supplementary file 1 — Full interview schedule. (DOCX 51 kb) [file 12888_2018_1722_MOESM1_ESM.docx]

# R09308 – National Survey of Community Health

## Introduction

A1. Good (morning/afternoon/evening). My name is (….) and I am calling from Roy Morgan Research on behalf of a research team at the Centre for Mental Health at the University of Melbourne. Researchers at the University are conducting a major study on what Australian adults understand about recognising and assisting an individual in severe distress.

[STATE THE FOLLOWING IF RESPONDENT ASKS HOW YOU GOT THEIR NUMBER: Your telephone number has been chosen at random from all possible telephone numbers in your area. We find that this is the best way to obtain a representative sample of people across Australia.]

A2. For the study, we’d like to interview the person living in this household who is 18 or older who had the last birthday. Would that be you?

A1. [REINTRODUCE IF NECESSARY: Good (morning/afternoon/evening). My name is (….) and I am calling from Roy Morgan Research on behalf of a research team from the Centre for Mental Health at the University of Melbourne. Researchers at the University are conducting a major study on what Australian adults understand about recognising and assisting an individual in severe distress]

A3. For this study, we are interested in talking to people aged 18 or over. Can I check, are you aged 18 years or over?

A4. The interview takes between 10 and 25 minutes, depending on your answers. If you agree to participate, you will be asked a series of questions about recognising and assisting an individual in severe distress.

Before we continue, I need to tell you a few details about your participation.

The study is funded by *beyondblue*. Participation is completely voluntary and, even if you agree to be interviewed, you can withdraw at any time.

All data is kept confidential within the limits of the law.

The information gained from this study may be used in future research studies, and if so, your confidentiality will be respected in the same way.

If you have any questions about the study, I can direct you to a website with more information or I can give you the number of one of the researchers or for the University’s ethics officer. Also, if at any time you would like phone numbers for organisations that support people in distress, please let me know. I will give these to you at the end of our call, for future reference.

Do you have any questions about the study, or would you like to go ahead?

## Demographics

### B1. Age categories

**NUMERIC**

B1.1 How old are you? [ENTER AGE] or Code 99/Refused

TERMINATE IF LESS THAN 18 YEARS

**ASK IF CODE 99 AT B1.1**

**SINGLE**

B1.2 Would you mind telling me which age group do you belong to?

1. Less than 18 years (TERMINATE)
2. 18-20
3. 21-25
4. 26-30
5. 31-35
6. 36-39
7. 40-44
8. 45-49
9. 50-54
10. 55-59
11. 60-64
12. 65-69
13. 70-74
14. 75+
15. (Refused)

[IF REFUSES, TERMINATE INTERVIEW]

### B2 Gender

### ASK ALL

### SINGLE

B2. And could you please tell me your gender?

1. Male
2. Female
3. Other
4. Refused - **TERMINATE**

## Presentations of Vignettes

C1 Now I am going to ask you about the difficulties and challenges of a person I will call (John/Jenny). (John/Jenny) is not a real person, but there are people like (him/her).

If at any time you would like me to read out the scenario again, let me know.

**RANDOMISE C2 to C7 – EACH RESPONDENT GETS ASKED ONE OF C2 TO C7**

C2 Imagine John is someone you know well. You have noticed that he has been looking unusually sad and miserable for the last few weeks. He looks tired all the time, and he tells you he has been having trouble sleeping nearly every night. He is also very withdrawn, talking very little at work and avoiding social situations. When you ask him about what is going on, John tells you that he and his partner have separated, and he is having financial problems.

C3 Imagine Jenny is someone you know well. You have noticed that she has been looking unusually sad and miserable for the last few weeks. She looks tired all the time, and she tells you she has been having trouble sleeping nearly every night. She is also very withdrawn, talking very little at work and avoiding social situations. When you ask her about what is going on, Jenny tells you that she and her partner have separated, and she is having financial problems.

C4 Imagine John is someone you know well. You have noticed that he has been looking unusually sad and miserable for the last few weeks. He looks tired all the time, and he tells you he has been having trouble sleeping nearly every night. He is also very withdrawn, talking very little at work and avoiding social situations. When you ask him about what is going on, John tells you that he and his partner have separated, and he is having financial problems. John says he feels he will never be happy again and believes his family would be better off without him.

C5 Imagine Jenny is someone you know well. You have noticed that she has been looking unusually sad and miserable for the last few weeks. She looks tired all the time, and she tells you he has been having trouble sleeping nearly every night. She is also very withdrawn, talking very little at work and avoiding social situations. When you ask her about what is going on, Jenny tells you that she and her partner have separated and she is having financial problems. Jenny says she feels she will never be happy again and believes her family would be better off without her.

C6 Imagine John is someone you know well. You have noticed that he has been looking unusually sad and miserable for the last few weeks. He looks tired all the time, and he tells you he has been having trouble sleeping nearly every night. He is also very withdrawn, talking very little at work and avoiding social situations. When you ask him about what is going on, John tells you that he and his partner have separated, and he is having financial problems. John says he feels he will never be happy again and believes his family would be better off without him. You run into a friend of John’s. She tells you that John told her he feels desperate and has been thinking of ways to end his life.

C7 Imagine Jenny is someone you know well. You have noticed that she has been looking unusually sad and miserable for the last few weeks. She looks tired all the time, and she tells you she has been having trouble sleeping nearly every night. She is also very withdrawn, talking very little at work and avoiding social situations. When you ask her about what is going on, Jenny tells you that she and her partner have separated, and she is having financial problems. Jenny says she feels she will never be happy again and believes her family would be better off without her. You run into a friend of Jenny’s. He says that Jenny told him she feels desperate and has been thinking of ways to end her life.

## Intentions and Confidence

### D1. Intentions: Open-ended

**OPEN TEXT**

D1. Remembering {John/Jenny} is someone you know well.

What, if anything, would you do? Anything else?

INTERVIEWER NOTE – RECORD VERBATIM, PROBE FULLY

## D2. Confidence

**SINGLE**

D2. How confident would you feel supporting {John/Jenny}?

Would you be…… READ OUT

1. Not at all confident
2. Not confident
3. Neither confident nor not confident
4. Confident
5. Extremely confident]

### D3. Intentions

Some of the items in the following question might repeat something you have told me already, which is fine.

How likely is it that you would take the following actions with {John /Jenny}?

[PROGRAMMER NOTE – PROGRAM AS GRID WITH THE FOLLOWING RESPONSE SCALE FOR EACH ITEM]

Very unlikely

Unlikely

Neither likely nor unlikely

Likely

Very likely

D3. i Ask about how {he/she} is feeling

D3. ii Listen to {John’s/Jenny’s} problems without judgement

D3. iii Remind {him/her} what {he/she} has going for {himself/herself}

D3. iv Ask how you can help

D3. v Try to solve {John’s/Jenny’s} problems

D3 vi Reassure {John/Jenny} that you know exactly how badly {he/she} feels

D3. vii Help make an appointment with a health professional – for example a GP or counsellor

D3. viii Call a crisis line – for example, Lifeline

D3. ix Go to an appointment with a professional with {him/her} – for example a GP

D3. x Ask if {he/she} has been thinking about killing {himself/herself}

D3. xiii If {John/Jenny} told me {he/she} was thinking about killing {himself/herself}, I would try to make {him/her} understand that suicide is wrong.

D3. xiv If {John/Jenny} told me {he/she} was thinking about killing {himself/herself}, I would ask if {he/she} has a means to kill {herself/himself} – for example, pills or a weapon

D3. xv If {John/Jenny} told me {he/she} was thinking about killing {himself/herself} I would listen to why {he/she} wants to die.

D3. xi I would tell {him/her} how much it will hurt {his/her} family and friends if {he/she} were to kill {himself/herself}

D3. xii I would ask if {he/she} has a plan for suicide – for example a date or how they will die.

### Barriers and enablers

**OPEN TEXT**

E1. What might stop you from supporting John/Jenny? Anything else?

INTERVIEWER NOTE: RECORD VERBATIM, PROBE FULLY

**OPEN TEXT**

E2. What might assist you in supporting John/Jenny? Anything else?

INTERVIEWER NOTE – RECORD VERBATIM, PROBE FULLY

## Actual helping behaviour

### F1. Has someone close to you experienced a similar level of distress?

**SINGLE**

F1. In the last 12 months, has anyone in your family or close circle of friends experienced a similar level of distress to {John/Jenny}? DO NOT READ

INTERVIEWER NOTE: EXCLUDES RESPONDENT THEMSELF

1. Yes [GO TO F2]
2. No [GO TO G]
3. Don't know [GO TO G]
4. Refused [GO TO G]

**F2. Helping someone**

**ASK IF KNOWS SOMEONE LIKE JOHN/JENNY (Code 1 at F1)**

**SINGLE**

F2.1. Did just one of your family or close friends experience this level of distress in the last 12 months, or more than one? DO NOT READ

1. One [GO TO F2.3]
2. More than one [GO TO F2.2]
3. Don't know [GO TO G]
4. Refused [GO TO G]

**ASK IF KNEW MORE THAN ONE PERSON (Code 2 at F2.1)**

F2,2 Because you know more than one family member or close friend experiencing a similar level of distress, for the next few questions, I want you to think about the one you know BEST.

**ASK IF KNOWS A PERSON LIKE JOHN/JENNY (Code 1 at F1)**

**SINGLE**

F2.3 Did you do anything to support this person? DO NOT READ

1. Yes [GO TO F2.4]
2. No [GO TO G]
3. Don’t know [GO TO G]
4. Refused [GO TO G]

**ASK IF TRIED TO HELP SOMEONE LIKE JOHN/JENNY (Code 1 at F2.3)**

**OPEN TEXT**

F2.4 What did you do? Anything else?

INTERVIEWER NOTE: RECORD VERBATIM, PROBE FULLY

**F2.5 SPECIFIC ACTIONS**

Again, items in the following question might repeat something you have already told me, but that is fine.

F2.5 Did you do any of the following to try to support that person?

PROGRAMMER NOTE: PROGRAM AS GRID, WITH YES/NO RESPONSE FOE EACH ITEM

F2. i Asked about how they were feeling

F2. ii Listened to their problems without judgement

F2. iii Reminded them what they have going for them

F2. iv Asked how you could help

F2. v Tried to solve their problems

F2 vi Reassured them that you know exactly how badly they feel

F2. vii Helped make an appointment with a health professional – for example a GP or counsellor

F2. viii Called a crisis line – for example, Lifeline

F2. ix Went to an appointment with a professional with them – for example, a GP

F2. x Asked if they had been thinking about killing themselves

**F2.6 IF PERSON WAS SUICIDAL**

F2.6. When you were supporting that person, did you ever find out or suspect they might be thinking about suicide? ? DO NOT READ

- 1. YES [GO TO F2.6.i]
  2. NO [GO TO G]
  3. NOT SURE [GO TO F2.6.i]
  4. REFUSED [GO TO G]

**ASK IF PERSON SAID WERE THINKING OF SUICIDE (CODE 1 at F2.6)**

When you found out or suspected they were thinking about suicide, did you do any of the following?

**ASK IF NOT SURE IF PERSON SAID WERE THINKING OF SUICIDE (CODE 3 at F2.6)**

Did you do any of the following?

PROGRAMMER NOTE: PROGRAM AS GRID WITH YES/NO RESPONSE FOR EACH ITEM

F2.6.i Tell them how much it would hurt their family and friends if they were to kill themselves

F2.6.ii Ask if they had a plan for suicide – for example a date or how they will die

F2.6.iii Try to make them understand that suicide is wrong

F2.6.iv Asked if they had a means to kill themselves – for example, pills or a weapon

F2.6. v Listened to why they wanted to die

## Own suicidal thoughts

Feelings like John’s/Jenny’s are quite common. The previous set of questions asked about other people you know who have had difficulties like John’s/Jenny’s. Now I want to ask you some questions about your own experiences.

### G1. Own suicidal thoughts and behaviours

**ASK ALL**

**SINGLE**

G1.1 Has there been a time in the last 12 months that you thought of taking your own life, even if you would not really do it? DO NOT READ

1. Yes [GO TO G 1.2]
2. No [GO TO I]
3. Don’t know [GO TO I]
4. Refused [GO TO I]

**ASK IF HAD SUICIDAL THOUGHTS (Code 1 at G1.1)**

**SINGLE**

G1.2 Has there been a time in the last 12 months when you reached the point where you seriously considered taking your own life, or perhaps made plans how you would go about doing it? DO NOT READ

1. Yes [GO TO G1.3]
2. No [GO TO G2]
3. Don’t know [GO TO G2]
4. Refused [GO TO G2]

**ASK IF SERIOUSLY CONSIDERED SUICIDE (Code 1 at G1.2)**

**SINGLE**

G1.3 In the last 12 months, have you made an attempt to take your life? DO NOT READ

1. Yes
2. No
3. Don’t know
4. Refused

### G2. Professional help-seeking

**ASK IF ASK IF HAD SUICIDAL THOUGHTS (Code 1 at G1.1)**

**SINGLE**

G2. Did you receive help from a health professional for your suicidal thoughts? DO NOT READ

1. Yes
2. No

## Other person help you

### H1. Receiving help

**SINGLE**

H1 Did anyone, other than a professional, try to help you when you were having suicidal thoughts?

1. Yes [GO TO H2]
2. No [GO TO I]
3. Don’t know [GO TO I]
4. Refused [GO TO I]

### H2. Type of help

**ASK IF SOMEONE OFFERED HELP (Code 1 at H1)**

If more than one person tried to help you, other than a professional, think of the person that helped you the most

**OPEN TEXT**

H2.1 What did this person do? Anything else?

INTERVIEWER NOTE: RECORD VERBATIM, PROBE FULLY

**GRID**

H2.2 Please answer yes or no as to whether that person offered you the following types of help or action:

PROGRAMMER NOTE: PROGRAM AS GRID WITH YES/NO RESPONSE FOR EACH ITEM

H2. i Asked you about how you were feeling

H2. ii Listened to your problems without judgement

H2. iii Reminded you what you had going for you.

H2. iv Asked how they could help

H2. v Tried to solve your problems

H2 vi Reassured you they knew exactly how badly you were feeling

H2. vii Helped make an appointment with a health professional – for example a GP or counsellor

H2. viii Called a crisis line, for example Lifeline

H2. ix Went with you to an appointment with a professional – for example a GP

H2. x Asked you if you were thinking about killing yourself

H2. xi Told you how much it would hurt your family and friends if you killed yourself.

H2. xii Asked you if you had a plan for suicide – for example a date or how you will die

H2. xiii Tried to convince you that suicide is wrong

H2. xiv Asked if you had a means to kill yourself - for example, pills or a weapon

H2. xv Listened to why you wanted to die.

### H3. Helpfulness

**SINGLE**

H.3.1. Overall, how helpful did you find what that person did when you were having suicidal thoughts? Was it…. READ OUT

1. Not at all helpful
2. Not helpful
3. Neither helpful nor unhelpful
4. Helpful
5. Extremely helpful

**OPEN TEXT**

H.3.2 What was the most helpful thing they did for you? ]

INTERVIEWER NOTE: RECORD VERBATIM

**OPEN TEXT**

H.3.3 What was the most unhelpful thing they did for you?

INTERVIEWER NOTE: RECORD VERBATIM

## I. Attitudes

**ASK ALL**

**GRID**

Please state how much you agree or disagree with the statements in this section using the following scale:

strongly agree

agree

neither agree nor disagree

disagree

strongly disagree

PROGRAMMER NOTE: PROGRAM AS GRID, RANDOMISE ORDER OF QUESTIONS

I1. There is a risk that asking someone about suicide will make them start thinking about it.

I2. Helpful treatments exist for many of the conditions that lead to suicide.

I3. Suicide happens without warning.

I4. If someone wants to kill themselves, it is his or her own business and we should not interfere.

I5. People who make suicidal threats rarely kill themselves.

I6. Once a person has made up his or her mind about suicide, no one can stop him or her.

I7. Helping a person at risk of suicide requires the skill of a professional

I8. It is a human duty to try to stop someone from killing themselves.

I9. Suicidal people are selfish.

I10. Most suicides occur without any sign.

I11. I am comfortable talking to someone I know well about suicide.

I12. There is a risk of making the situation worse if I try to help someone who is thinking about suicide.

## Exposure to suicide

The following questions ask about any one you know who has died by suicide.

### J1. Exposure to any suicide

**SINGLE**

J1.1 Do you know anyone who has died by suicide? DO NOT READ

1. Yes [GO TO J1.2]
2. No [GO TO K]
3. Don’t know [GO TO K]
4. Refused [GO TO K]

**ASK IF KNOWS SOMEONE WHO DIED BY SUICIDE (Code 1 at J1.1)**

**NUMERIC (3 digits)**

J1.2 How many people do you know who died by suicide?

INTERVIEWER NOTE: Record Number. Code 999 for refused, 998 for don’t know.

**ASK IF MORE THAN ONE PERSON (2 to 999 at J1.2)**

Thinking of the person you knew best who died by suicide….

### J3. Closeness to someone who died by suicide

**ASK IF KNOWS SOMEONE WHO DIED BY SUICIDE (Code 1 at J1.1)**

SINGLE

J3.1 On a scale of 1 to 5, where one means ‘not close’ and five means ‘very close’, how close was your relationship to that person?

1. Not close


5. Very close

### J4. Effect of the suicide

**NUMERIC (4 Digits)**

J4.1 In what year did that death occur?

INTERVIEWER NOTE: IF CAN’T REMEMBER EXACT YEAR, ASK FOR THEIR BEST GUESS

**SINGLE**

J4.2 Thinking about the effect of that person’s suicide on your life, which response is closest to your experience? Did it have…

- 1. Little effect on your life
  2. It had somewhat of an effect on you but did not disrupt your life
  3. It disrupted your life for a short time
  4. It disrupted your life in a significant or devastating way, but you no longer feel that way
  5. The death had a significant or devastating effect on your life that you still feel.

## K. Formal training in suicide prevention

### K1. Professional experience with suicide prevention

**ASK ALL
SINGLE**

K1.1 Have you ever had a job that involved providing treatment or services to a person experiencing distress like {John’s/Jenny’s}? DO NOT READ

1. Yes [GO TO K1.2]
2. No [GO TO K2.1]
3. Don’t know [GO TO K2.1]
4. Refused [GO TO K2.1]

**ASK IF HAD SUCH A JOB (Code 1 at K1.1)**

**MULTIPLE**

K1.2 Could you tell me the type of job? [CHOOSE FROM LIST OR SPECIFY IF ‘OTHER’. CODE AS MANY AS APPLY]

INTERVIERWER NOTE: PROBE AS NECESSARY AND CODE, OR RECORD AS VERBATIM IN ‘OTHER’

1. Nurse
2. General practitioner
3. Psychiatrist
4. Emergency department doctor
5. Psychologist
6. Social worker
7. Counsellor
8. Welfare worker/ Welfare support worker
9. Ambulance officer / Paramedic
10. Youth worker
11. Police
12. Other (Specify)
13. (Don't know)
14. (Refused)

### K2. Training in suicide prevention

**ASK ALL
SINGLE**

K2.1 Have you ever completed any training or course in how to help someone who is suicidal?

INTERVIEWER NOTE: PROBE AS NECESSARY AND CODE, OR RECORD AS VERBATIM IN ‘OTHER’

1. Yes, professional training (refers to training received in relation to becoming a professional in previous question): Please specify.
2. Yes, other: Please specify
   1. Mental health first aid
   2. ASIST
   3. QPR (Question, Persuade, Refer)
   4. Other, please specify
3. No
4. Don’t know
5. Refused

### L. EXPOSURE TO SUICIDE PREVENTION MESSAGES

**SINGLE**

L1. Do you recall hearing or seeing any advertising or media messages in the last 12 months, relating to helping people who are thinking about suicide? DO NOT READ

1. Yes [GO TO L2]
2. No [GO TO M]
3. Don’t know [GO TO M]
4. Refused [GO TO M]

**ASK IF RECALLED MEDIA (Code 1 at L1)**

**OPEN TEXT**

L2. What was the main message of the advertising or media message that you heard/saw that you remember most clearly?

INTERVIEWER NOTE: RECORD VERBATIM

**MULTIPLE**

L3. And where did you see or hear this advertising or media message(s)? Anywhere else?

INTERVIEWER NOTE: PROBE AND CODE TO ALL CHANNELS MENTIONED> IF DON’T KNOW WHERE TO CODE, RECORD IN DETAIL AT ‘OTHER’

Advertising channels

1. TV advertising
2. Online advertising (e.g.: banner advertising, video pre-rolls, catch-up TV)
3. Social media (e.g.: via Facebook, Twitter, Instagram)
4. Radio advertising
5. Online streaming services (e.g.: Spotify, Pandora)
6. Newspaper/magazine advertising
7. Outdoor advertising (Posters/billboards)

Non-advertising channels

1. TV program (not advertising, e.g.: news, current affairs, documentary)
2. Article in a newspaper or magazine (not advertising)
3. Radio broadcast (not advertising e.g. talkback or panel discussion)
4. Online article (eg: newspaper, blogs)
5. Online video (e.g.: Youtube, Vimeo)
6. Friends/family, colleagues
7. Other [SPECIFY]

### M. ADDITIONAL DEMOGRAPHICS

### Now, a few questions to help ensure we have spoken with a broad range of people

### M1. Marital status

**ASK ALL**

**SINGLE**

M1 What is your current marital status? READ OUT

1. Never married
2. Married or de facto
3. Separated, divorced or widowed
4. Refused DO NOT READ

### M2. Postcode where you normally live

**NUMERIC (4 Digits)**

M2.1 What is the post code of the place you usually live?

1. Enter Postcode
2. Don’t know
3. Refused

**ASK IF DON’T KNOW POSTCODE**

M2.2 Well could you please tell me the name of the suburb or town in which you live?

PROGRAMMER NOTE: UINSERT LOOK UP TABLE OF LOCALITIES

### M3. Aboriginal and Torres Strait Islander

**ASK ALL
SINGLE**

M3.1 Are you of Aboriginal or Torres Strait Islander origin? DO NOT READ

1. Yes [GO TO L3.2]
2. No
3. (Refused)

**ASK IF ABORIGINAL OR TORRES STRAIT ISLANDER
SINGLE**

M3.2 Are you Aboriginal, Torres Strait Islander, or both? DO NOT READ

1. Aboriginal
2. Torres Strait Islander
3. Both

### M4. Country of Birth

**ASK ALL
SINGLE**

In which country were you born? DO NOT READ

1. Australia
2. UK
3. New Zealand
4. Italy
5. Greece
6. China
7. Vietnam
8. Other (Specify)
9. Don’t know
10. Refused

### M5. Language other than English

**SINGLE**

M5.1 Do you mainly speak a language other than English at home? DO NOT READ

1. No, English only or mainly[GO TO L6]
2. Yes [GO TO L5.2]

**IF MAINLY SPEAKS L.O.T.E AT HOME (Code 1 at M5.1)**

**SINGLE**

M5.2 What is the language other than English you speak most at home? DO NOT READ

1. Italian
2. Greek
3. Cantonese
4. Mandarin
5. Arabic
6. Vietnamese
7. German
8. Spanish
9. Tagalog (Filipino)
10. Other (Specify)
11. Don’t know
12. Refused

### M6. Highest level of education

**ASK ALL
SINGLE**

M6 What is the highest level of education you have completed?

INTERVIEWER NOTE: READ OUT IF NECESSARY

1. Year 9 or lower
2. Year 10
3. Year 11
4. Year 12
5. Trade certificate/apprenticeship
6. Other certificate
7. Associate or undergraduate diploma
8. Bachelor's degree or higher
9. Other (Specify)
10. Don’t know
11. Refused

### M7. People in the household

**NUMERIC (2 digits)**

M7 How many people live in your household, including yourself?

INTERVIEWER NOTE: CODE 99 FOR REFUSED, CODE 98 FOR DON’T KNOW

ASK IF MOBILE SAMPLE

M8. Do you live in a home that also has a landline telephone?

ASK IF LANDLINE SAMPLE

M9. Do you personally have a mobile phone?

### ASK ALL

### N. RECONTACT

The researchers are hoping to carry out a follow-up study on this topic in about a year’s time. If you agree to re-contact now you are not obliged to participate in the future; participation in any future studies is completely voluntary.

### N1. Interest in future participation

**SINGLE**

N1 Would you be interested in being contacted again in the future to answer a similar set of questions? DO NOT READ

1. Yes [GO TO N2]

2. No [GO TO O]

### N2. First name

**ASK IF WILLING TO BE CONTACTED (Code 1 at N1)**

**OPEN TEXT**

N2 Could you please tell me your first name? [SPECIFY]

### N3. Last name

**OPEN TEXT**

N3 And your last name? [SPECIFY]

### N4. Email address

**SINGLE**

N4 Do you have an email address?

- 1. [SPECIFY EMAIL ADDRESS]
  2. No/refused

### N5. Prefered number

**SINGLE**

**N5** Is the telephone number you are currently on your preferred number?

1. Yes
2. No [ENTER NEW NUMBER INCLUDING AREA CODE] [GO TO N7]

### N6. Alternative number

### ASK IF CURRENTLY ON PREFERRED NUMBER (Cod 1 at N5)

**SINGLE**

N6 And do you have an alternative number we could contact you on next time?

1. Yes [ENTER NEW ALTERNATIVE NUMBER (INCLUDE AREA CODE)]
2. No

### N7. Alternative person to call

**ASK IF WILLING TO BE CONTACTED (Code 1 at N1)**

**SINGLE**

In case we are unable to contact you with these contact details, is there another person we could contact who will be able to get in touch with you?

1. Yes
2. No

**ASK IF WILLING TO PROVIDE ALTERNATIVE CONTACT (Code 1 at N7)**

**SINGLE**

N7.1 Could you please provide a phone number or email address for that person?

1. Yes (RECORD)
2. No/Refused

### O. CONTACTS FOR DISTRESS

**ASK ALL**

**SINGLE**

I understand that some of these questions may have been hard for you to talk about, but we really value and appreciate your help today. If you would like to talk to someone about any issues you might be facing or if you would like to talk to anyone on the research team about how the questions affected you, I will give you some contact numbers that you can write down.

1. Yes [PROVIDE NUMERS BELOW AS NEEDED]
2. Refused/Not required [GO TO P]

*beyondblue* support line (24 hours): 1300 22 4636

Lifeline: 131114 (24 hours)

headspace (ages 18 to 25 only): 1800 650 890

Suicide call-back service (only for those considering suicide, caring for someone at risk of suicide, or bereaved by suicide): 1300 659 467

(MELB UNIVERSITY LEAD RESEARCHER)

Professor Jane Pirkis, Chief Investigator

The University of Melbourne

PH: 03 8344 0647

EM: [j.pirkis@unimelb.edu.au](mailto:j.pirkis@unimelb.edu.au)

(MELBOURNE UNIVERSITY ETHICS OFFICER: IF CONCERNED ABOUT THE ETHICAL CONDUCT OF THE STUDY )

PH: 03 8344 2073

### P. CLOSE

Thanks for participating in this survey. Just in case you missed it, my name is (……) calling from Roy Morgan Research on behalf of the Centre for Mental Health at the University of Melbourne. The results of this research will be made available on the beyondblue website once it is complete ([www.beyondblue.org.au](http://www.beyondblue.org.au))
